# Successful prevention of exacerbation of thrombocytopenia in a pregnant patient with idiopathic thrombocytopenic purpura by anticoagulation treatment

**DOI:** 10.1186/s12884-015-0482-7

**Published:** 2015-02-26

**Authors:** Michi Hisano, Nobuhiro Tsukada, Haruhiko Sago, Koushi Yamaguchi

**Affiliations:** Center of Maternal-Fetal, Neonatal and Reproductive Medicine, National Center for Child Health and Development, 2-10-1 Okura, Setagaya-ku, 157-8535 Tokyo Japan; Division of Hematology, Japanese Red Cross Medical Center, 4-1-22 Hiroo, Shibuya-ku, 150-8935 Tokyo Japan

**Keywords:** Idiopathic thrombocytopenic purpura, Pregnancy, Thrombocytopenia, Anticoagulation

## Abstract

**Background:**

Corticosteroid or intravenous immunoglobulin is used in the management of idiopathic thrombocytopenic purpura during pregnancy.

**Case presentation:**

A patient with idiopathic thrombocytopenic purpura had a previous history of interrupted pregnancy due to severe thrombocytopenia, and was unresponsive to high doses of corticosteroids and intravenous immunoglobulin. Immediately following pregnancy, our patient had a marked elevation in plasma levels of fibrinogen degradation products, D-dimer, and platelet factor 4, with a decrease in platelets, suggesting platelet activation and thrombogenesis. Combined treatment with an anticoagulant agent could prevent exacerbation of thrombocytopenia throughout pregnancy. Although the underlying causes leading to the series in her pregnancy course were uncertain, there were notable serological abnormalities, such as weakly positive antinuclear antibody and anti-U1-RNP antibody.

**Conclusion:**

When thrombocytopenia rapidly develops in patients with idiopathic thrombocytopenic purpura immediately following pregnancy, the possibility of a thrombogenic state and differential diagnosis, including antiphospholipid syndrome and collagen vascular disease, should be considered. Treatment with an anticoagulant agent might then be appropriate.

## Background

Thrombocytopenia is often experienced in pregnancy, affecting up to 10% of all pregnancies [1,2]. Idiopathic thrombocytopenic purpura (ITP) is a common cause of thrombocytopenia in the first and second trimesters. The pathogenesis of ITP is related to the production of anti-platelet antibodies, resulting in accelerated clearance and destruction of opsonized platelets by the reticuloendothelial system. Because anti-platelet antibodies also target antigens on megakaryocytes, suppressed platelet production might be the mechanism causing thrombocytopenia in ITP [3]. In general, corticosteroids are the first-line treatment for pregnant women with ITP, and intravenous immunoglobulin (IVIG) is used to rapidly raise the platelet count [4-6]. The use of rituximab and thrombopoietic agents during pregnancy for ITP has been avoided because of limited information on their safety and clinical effects. In our ITP case, combined treatment with an anticoagulant agent from the early weeks of pregnancy was useful for reducing the progression of thrombocytopenia.

## Case presentation

A 28-year-old woman with ITP visited our hospital with the desire to have children. ITP had been already diagnosed by a prior history of bleeding and a low platelet count, and other possible causes of thrombocytopenia were excluded based on physical and bone marrow examinations. Recently, she had been hospitalized for severe thrombocytopenia. She had a platelet count of 3 × 10^9^/L and subcutaneous bleeding immediately following pregnancy at 6 weeks’ gestation, despite maintaining the platelet count at 30–90 × 10^9^/L with 3–5 mg/day of prednisolone (PSL) before pregnancy. The patient’s platelet count could not be maintained at higher than 30 × 10^9^/L, even with two treatments of IVIG (0.4 g/kg/day for 5 days) and intravenous pulses of methylprednisolone (0.5 g/day for 4 days) followed by oral PSL (30 mg/day). Her attending physician decided to end her pregnancy with platelet transfusion for reasons of maternal safety at 10 weeks’ gestation. To ensure subsequent pregnancies, we controlled the platelet count at pre-pregnancy at a set point of higher than 100 × 10^9^/L with 17.5 mg/day of PSL. The patient then became pregnant for the second time.

**Figure 1:**
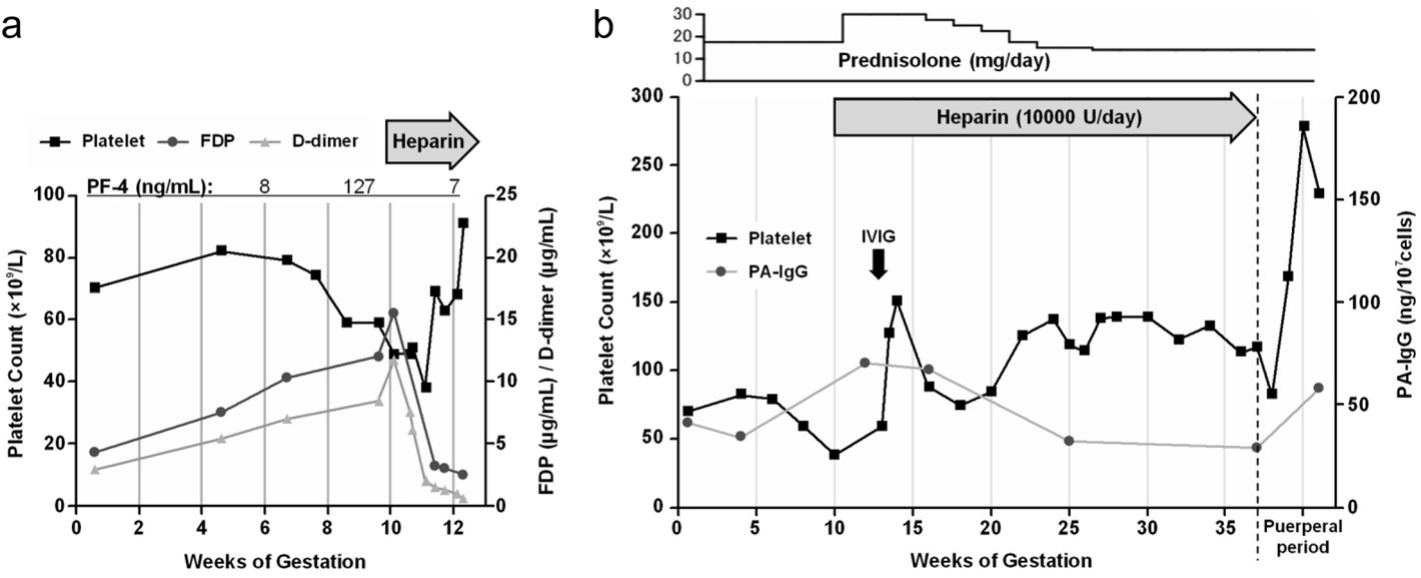
**Clinical course. a** Changes in platelet count and levels of FDP, D-dimer, and PF-4 before and after treatment with unfractionated heparin (10,000 U/day). **b** Medications and changes in platelet count and PA-IgG values in the pregnant and puerperal periods.

Initial laboratory studies at the first visit after pregnancy showed a white blood cell count of 4.83 × 10^9^/L, hemoglobin of 140 g/L, mild thrombocytopenia with a platelet count of 70 × 10^9^/L, and high levels of fibrinogen degradation products (FDP) (4.3 μg/mL; normal range, <4) and D-dimer (2.9 μg/mL; normal range, <1). The results of other blood coagulation tests were as follows: prothrombin time/international normalized ratio of 0.96, activated partial thromboplastin time of 23.4 s, fibrinogen level of 2.56 g/L, antithrombin III activity of 92.1%, protein C activity of 89% (normal range, 64–146%), protein C antigen level of 81% (normal range, 70–150%), protein S activity of 52% (normal range, 60–150%), protein S antigen level of 75% (normal range, 65–135%), and platelet factor 4 (PF-4) level of 8 ng/mL (normal range, <20 ng/mL). Immunological analysis showed a weakly positive antinuclear antibody titer of 1:80, anti-DNA antibody was 2.0 IU/mL (normal range, <6 IU/mL), anti-U1-RNP antibody was 121 U/mL (normal range, <10 IU/mL), CH50 was 45.1 U/mL, C3 was 84 mg/dL, C4 was 24 mg/dL, and platelet-associated IgG (PA-IgG) was 59 ng/10^7^cells (normal range, <46 ng/10^7^cells). Lupus anticoagulant was 1.03 (normal range, <1.3) and antiphospholipid antibodies, including anti-cardiolipin IgG antibodies, anti-β_2_-glycoprotein I antibodies, and anti-prothrombin antibodies, were not detected. Immediately following pregnancy, an elevation in FDP and D-dimer levels, with a decrease in platelets was observed (Figure 1a). Thrombocytopenia and a fibrinolytic state became significant with the date of gestation and the level of PF-4 was increased (127 ng/mL) at 9 weeks’ gestation. This suggested that a rapid decrease in platelet count after pregnancy might be associated with platelet activation and thrombogenesis, as well as with antibody-mediated destruction of platelets. Our patient had no clinical evidence of arterial and venous thrombosis. No significant aberrations in platelet aggregation tests (collagen, 67%; ristocetin, 78%; epinephrine, 68%; adenosine diphosphate, 70%), von Willebrand factor antigen level (362%; normal range, 20–155%), von Willebrand factor activity (388%; normal range, 60–170%), ADAM13 activity (158%; normal range, 70–120%), and factor VIII activity (200%; normal range, 62–145%) were observed. Anticoagulation therapy was performed by continuous unfractionated heparin injection (10,000 U/day), and the dose of PSL was increased to 30 mg/day at 10 weeks’ gestation. We used additional IVIG (0.4 g/kg/day for 5 days), albeit early in the course of the disease, and also expected to minimize the dose of corticosteroids in the first trimester. The platelet count recovered from 40 × 10^9^ /L to 90 × 10^9^ /L for 3 weeks. The laboratory findings in the second trimester were as follows: PA-IgG was 32 ng/10^7^cells, platelet antibody-secreting B cells for GPIIb/IIIa antigen by ELISPOT assay was 2.3 × 10^6^ cells/mL (normal range, <2.0 × 10^6^ cells/mL), the reticuloplatelet ratio was 3.7% (normal range, 0.7–3.0%), thrombopoietin was 0.44 pg/mL (normal range, <300 pg/mL), and PF-4 was 13 ng/mL. The dose of PSL was tapered below 15 mg daily and heparin treatment was continued throughout pregnancy. The platelet count was maintained above 100 × 10^9^/L (Figure 1b), and she delivered a 2500-g boy vaginally at 37 weeks’ gestation without any hemorrhage. Infant Apgar scores were 9 and 10 at 1 and 5 min, respectively. Neonatal thrombocytopenia secondary to maternal ITP did not occur.

## Conclusions

The causes of pregnancy-specific thrombocytopenia are gestational thrombocytopenia, pre-eclampsia, HELLP syndrome, and acute fatty liver of pregnancy [1,2]. Gestational thrombocytopenia, which is the most common cause of thrombocytopenia in pregnancy (approximately 75% of cases), may be difficult to differentiate from ITP. However, gestational thrombocytopenia generally causes mild thrombocytopenia, usually >70 × 10^9^/L, from the mid-second or third trimester, and is not related to adverse events for the mother and newborn. In some cases, pregnancy might lead to worsening of thrombocytopenia in patients with ITP [4]. This may be caused by the effects of the hormonal milieu of pregnancy on the reticuloendothelial system, similar to those reported in autoimmune hemolytic anemia [7]. In our case, moderate thrombocytopenia appeared immediately following pregnancy. Therefore, we were concerned that the patient might become seriously ill again. The differences between a typical ITP course and that in the current patient with ITP were her laboratory findings, which were suggestive of a thrombogenic state; elevation of markers of intravascular coagulation; and platelet activation (FDP, D-dimer, and PF-4). The patient did not have any underlying disease that could be associated with disseminated intravascular coagulation. Overt disseminated intravascular coagulation scoring proposed by the International Society of Thrombosis and Hemostasis was 4 points. Antiphospholipid syndrome is a disorder that occurs with exacerbation in association with pregnancy, and is known to cause arterial and venous thrombosis, thrombocytopenia, complications in pregnancy (miscarriage, fetal loss, and pre-eclampsia), and livedo reticularis [8]. Antiphospholipid antibodies, especially anti-cardiolipin antibodies, anti-β_2_-glycoprotein I antibodies, and lupus anticoagulant, are associated with antiphospholipid syndrome, which is often observed in patients with systemic lupus erythematosus. In our case, antiphospholipid antibody and anti-DNA antibody were negative, but anti-U1-RNP antibody, characteristic of mixed connective tissue disease, and weakly positive antinuclear antibody were detected. However, the patient did not have clinical findings that suggested the presence of mixed connective tissue disease, including Raynaud phenomenon, swollen hands or puffy fingers, arthritis, myositis, and pulmonary involvement. We consider that the cause of thrombocytopenia in this patient may have been due to antibody-mediated destruction of platelets, as well as platelet activation and consumption following microvascular injury and thrombogenesis. In a previous pregnancy in our patient, artificial abortion had to be chosen because of severe thrombocytopenia, despite two treatments of IVIG and high doses of corticosteroids. Anticoagulant therapy using unfractionated heparin (10,000 U/day) helped to inhibit the progression of a decrease in platelet count and the dose of corticosteroids was reduced during pregnancy this time. Although we considered that it was reasonable to use aspirin as an antiplatelet agent for our patient’s clinical condition, in the event that thrombocytopenia became more severe, the patient would be at high risk for bleeding. We found it difficult to decide how long heparin treatment should be continued during pregnancy. The underlying causes leading to the series in our patient’s pregnancy course are uncertain. However, considering her laboratory findings, such as rapidly developing thrombocytopenia and a fibrinolytic state in the first trimester that manifested as antiphospholipid syndrome, we continued heparin treatment to prevent fetal loss in later pregnancy and maternal complications, such as thrombosis, preeclampsia, and HELLP syndrome. Notably, we also observed serological abnormalities, such as weakly positive antinuclear antibody and anti-U1-RNP antibody. Although clinical manifestations suggestive of collagen vascular disease have not appeared to date, we will continue to follow this patient. When thrombocytopenia rapidly develops in a patient with ITP immediately following pregnancy, and microvascular injury and thrombogenesis are suspected, markers of intravascular coagulation and platelet activation should be measured and collagen vascular disease should be screened for. Use of an anticoagulant agent might then be appropriate.

### Consent

Written informed consent was obtained from the patient for publication of this Case report.

## Abbreviations

ITP: Idiopathic thrombocytopenic purpura
IVIG: Intravenous immunoglobulin
PSL: Prednisolone
FDP: Fibrinogen degradation products
PF-4: Platelet factor 4
PA-IgG: Platelet-associated IgG
